# Complete plastome sequences of Lonicera L. species: implications for phylogeny and comparative analysis

**DOI:** 10.18699/vjgb-25-95

**Published:** 2025-10

**Authors:** S.S. Almerekova, M.M. Yermagambetova, D.Y. Yerbolatov, M.Y. Ishmuratova, Y.K. Turuspekov

**Affiliations:** Institute of Plant Biology and Biotechnology, Almaty, Kazakhstan; Institute of Plant Biology and Biotechnology, Almaty, Kazakhstan; Institute of Plant Biology and Biotechnology, Almaty, Kazakhstan; Karaganda Buketov University, Karaganda, Kazakhstan; Institute of Plant Biology and Biotechnology, Almaty, Kazakhstan Karaganda Buketov University, Karaganda, Kazakhstan

**Keywords:** Lonicera, Kazakhstan, next-generation sequencing, variable regions, DNA-barcoding markers, simple sequence repeats, Lonicera; Казахстан; секвенирование нового поколения; вариабельные регионы; маркеры ДНК-баркодирования; простые повторяющиеся последовательности, Lonicera, Казахстан, секвенирование нового поколения, вариабельные регионы, маркеры ДНК-баркодирования, простые повторяющиеся последовательности

## Abstract

Lonicera L. is one of the largest and economically significant genera in the family Caprifoliaceae Juss., with a controversial taxonomy. To contribute to its molecular taxonomy, we sequenced the plastomes of Lonicera species: Lonicera caerulea (two subspecies), L. tatarica, and L. micrantha – using next-generation sequencing technology and conducted a comparative analysis. Plastome sizes ranged from 153,985 bp in L. micrantha to 164,000 bp in L. caerulea subsp. pallasii, each containing 130 genes, including 85 protein-coding, 37 tRNA, and 8 rRNA genes. Five protein-coding (rps7, rps12, ndhB, ycf2, and ycf15), 7 tRNA (trnA-UGC, trnI-CAU, trnI-GAU, trnL-CAA, trnN-GUU, trnR-ACG, and trnV-GAC), and 4 rRNA (rrn4.5, rrn5, rrn16, and rrn23) genes were duplicated. Comparative analysis of Lonicera plastome boundaries revealed structural variations in L. caerulea subsp. altaica and L. caerulea subsp. pallasii, particularly in ndhA gene distribution. Three highly variable, two intergenic (ycf1-trnN-GUU and trnN-GUU-ndhF) and one genic (accD) region were identified. A total of 641 simple sequence repeats were detected in four plastomes. Phylogenetic analyses grouped Lonicera samples into two clades corresponding to subgenera Periclymenum and Chamaecerasus. In this study, the plastid genomes of two subspecies of L. caerulea and species L. micrantha were sequenced for the first time. The maximum likelihood tree derived from complete plastid genome sequences proved to be the most informative, showing a topology consistent with previous studies. The nucleotide sequences of variable regions (accD-ycf1-ndhF-trnN-GUU) demonstrate high potential for use in DNA barcoding and may serve as valuable molecular markers for species phylogenetic studies within the genus Lonicera.

## Abstract (ru)

Род Lonicera L. – один из крупнейших и экономически значимых в семействе Caprifoliaceae Juss., таксономия которого остается спорной. С целью внесения вклада в молекулярную таксономию данного рода мы секвенировали пластомы видов Lonicera – L. caerulea (два подвида), L. tatarica и L. micrantha – с использованием технологии секвенирования нового поколения и провели сравнительный анализ. Размеры пластомов варьировали от 153 985 п. н. у L. micrantha до 164 000 п. н. у L. caerulea subsp. pallasii; каждый пластом содержал 130 генов, включая 85 генов, кодирующих белок, 37 тРНК и 8 рРНК генов. Пять белок-кодирующих (rps7, rps12, ndhB, ycf2 и ycf15), 7 тРНК (trnA-UGC, trnI-CAU, trnI-GAU, trnL-CAA, trnN-GUU, trnR-ACG и trnV-GAC) и 4 рРНК (rrn4.5, rrn5, rrn16 и rrn23) гена были дуплицированы. Сравнительный анализ границ пластомов у видов Lonicera выявил структурные вариации у L. caerulea subsp. altaica и L. caerulea subsp. pallasii, особенно в распределении гена ndhA. Были идентифицированы три высоковариабельные области: две межгенные (ycf1-trnN-GUU и trnN-GUU-ndhF) и одна генная (accD). В четырех пластомах обнаружено всего 641 простая повторяющаяся последовательность (SSR). Филогенетический анализ сгруппировал образцы Lonicera в два клада, соответствующих подродам Periclymenum и Chamaecerasus. В настоящем исследовании впервые секвенированы пластидные геномы двух подвидов L. caerulea и вида L. micrantha. Древо, построенное методом maximum likelihood на основе полных последовательностей пластомов, оказалось наиболее информативным и демонстрировало топологию, согласующуюся с предыдущими исследованиями. Нуклеотидные последовательности вариабельных регионов (accD-ycf1-ndhF-trnN-GUU) обладают высоким потенциалом для использования в ДНК-баркодировании и могут служить ценными молекулярными маркерами для филогенетических исследований видов внутри рода Lonicera

## Introduction

Lonicera L. is the largest genus in the family Caprifoliaceae Juss., comprising approximately 140 species (Wang G.Q. et al., 2024), which are widely distributed across North America, Europe, Asia, and Africa (Donoghue et al., 2001; Wen, 2001). Lonicera species exhibit diverse constituents, including saponins, flavonoids, iridoids, phenolic acids, alkaloidal glycosides, etc. (Lin et al., 2008; Ali et al., 2013; Yang Q.R. et al., 2016; Ni, 2017). Moreover, it exhibits a range of biological activities, including antioxidant, anti-inflammatory, antiviral, anti-hepatoma, and hepatoprotective effects (Yoo et al., 2008; Park et al., 2012; Kong et al., 2017; Ge et al., 2018; Liu M. et al., 2020). Besides their biological activities, species of the Lonicera genus also hold significant ornamental value and are widely used in landscaping (Hayes, Peterson, 2020; Varlashchenko et al., 2021).

(Abdulina, 1999), two of which are listed in the Red Book of Kazakhstan (Baitulin, Sitpayeva, 2014). The species Lonicera caerulea subsp. altaica (Pall.) Gladkova, L. caerulea subsp. pallasii (Ledeb.) Browicz, L. tatarica L., and L. micrantha Trautv. ex Regel are widely distributed across Kazakhstan. According to the Plants of the World Online (https://powo. science.kew.org/) L. caerulea subsp. altaica is native to a vast range extending from Eastern Europe to Siberia and Mongolia, while L. caerulea subsp. pallasii is found in Northern and Eastern Europe, Siberia, and Central Asia. L. tatarica occurs naturally from Eastern Europe to Central Siberia and northeastern China; in contrast, L. micrantha is native to Kazakhstan. These species play a crucial role in the region’s floral biodiversity and are of particular ecological and conservation significance. Additionally, they possess medicinal properties and have been traditionally used in folk medicine for various therapeutic applications (Golubev et al., 2022; Boyarskikh, Kostikova, 2023; Taldybay et al., 2024). In Kazakhstan, Lonicera species have been studied using botanical (Ametov et al., 2016; Vdovina et al., 2024), phytochemical (Kushnarenko et al., 2016), and biochemical (Vdovina, 2019) assessments

The phylogenetic relationships within Lonicera remain incompletely resolved, presenting ongoing systematic challenges and requiring revisions to its classification (Wang G.Q. et al., 2024). Over time, various classification systems for Lonicera have been proposed (Maximowicz, 1877; Rehder, 1903; Nakai, 1938; Hara, 1983). According to A. Rehder (1903), Lonicera is divided into two subgenera, Chamaecerasus (L.) Rehder and Periclymenum (Mill.) Rehder; within the subgenus Chamaecerasus, it is further classified into four sections: Isoxylosteum Rehder, Isika DC., Coeloxylosteum Rehder, and Nintooa DC. H. Hara (1983) proposed a classification, which was based on the study by C.J. Maximowicz (1877), dividing Lonicera into subgenera Lonicera and Caprifolium (Mill.) Dippel, with further subdivision of subgenus Lonicera into four sections (Isika (Anderson) Rehder, Caeruleae (Rehder) Nakai, Lonicera and Nintooa (Sweet) Maxim) and five subsections (Purpurascentes, Monanthae, Isika, Bracteatae, and Rhodanthae). Later, P.S. Hsu et al. (1988) classified Lonicera into subgenera Chamaecerasus and Lonicera; further, subgenus Chamaecerasus was divided into four sections (Coeloxylosteum, Isika, Isoxylosteum, and Nintooa).

With advancements in molecular genetic technologies, numerous studies have focused on the phylogenetics of Lonicera. For instance, M. Nakaji et al. (2015) investigated the phylogenetic relationships among 23 Japanese Lonicera species using nucleotide sequences of five plastid non-coding regions (rpoB-trnC, atpB-rbcL, trnS-trnG, petN-psbM, and psbM-trnD). The findings support the fundamental validity of the classification by H. Hara (1983) of higher taxonomic groups for Japanese Lonicera species. M. Srivastav et al. (2023) conducted a phylogenetic analysis using restriction site-associated DNA sequencing (RADSeq). The RADSeqbased phylogenetic tree revealed that the Coeloxylosteum, Isoxylosteum, and Nintooa sections within subgenus Chamaecerasus were monophyletic, whereas the Isika section was found to be paraphyletic. Using nuclear ribosomal DNA cistron and plastid genome data, X.L. Yang et al. (2024) confirmed the paraphyly of section Isika and the monophyly of sections Coeloxylosteum, Isoxylosteum, and Nintooa within subgenus Chamaecerasus, aligning with the findings of M. Srivastav et al. (2023). All of the above-mentioned studies have contributed to the classification of the genus Lonicera. However, due to widespread hybridization and introgression, the precise taxonomy of Lonicera remains unresolved (Wang H.X. et al., 2020).The plastid is a vital organelle for photosynthesis in plants and possesses its own genome (Howe et al., 2003). The plastome is uniparentally inherited and highly conserved in gene content and organization (Howe et al., 2003). It ranges in size from approximately 120 to 160 kb and exhibits a quadripartite structure consisting of two identical inverted repeats (IR) and two single-copy regions: a large single-copy (LSC) region and a small single-copy (SSC) region (Palmer et al., 1988; Ruhlman, Jansen, 2014).

Advancements in high-throughput sequencing technologies have greatly facilitated plastid genome research, making it more accessible and enabling comprehensive genomic analyses. To date, only a few studies have been conducted on the comparative analysis of Lonicera plastid genomes. For example, seven plastid genomes (L. ferdinandi, L. hispida, L. nervosa, L. fragrantissima var. lancifolia, L. stephanocarpa, L. tragophylla, and L. japonica) (Liu M.L. et al., 2018) and three plastid genomes (L. japonica, L. similis, and L. acuminata) (Yang C. et al., 2023) have been comparatively analyzed. Recent studies have demonstrated that nucleotide sequences of the plastid genome can provide valuable insights for phylogenomic analysis (Luo et al., 2021; Zhao et al., 2023) taxonomic classification (Li Q., 2022; Oyuntsetseg et al., 2024), and species identification, utilizing plastome sequences as a super barcode (Chen X. et al., 2018; Zhang Z. et al., 2019). Moreover, plastid genome nucleotide sequences serve as a valuable resource for identifying species-specific genetic markers, such as DNA barcoding (Hong et al., 2022; Tang et al., 2022; Almerekova et al., 2024), microsatellite (Zhu M. et al., 2021; Yermagambetova et al., 2023), and single nucleotide polymorphism (SNP) markers (Dong et al., 2021). Therefore, we believe that sequencing and comparative analysis of Lonicera plastomes can contribute insights into the taxonomic classification and phylogenetic relationships of the genus.

In this study, we sequenced the plastid genomes of Lonicera species (L. caerulea subsp. altaica, L. caerulea subsp. pallasii, L. tatarica, and L. micrantha) using Illumina Next Generation Sequencing technology. Among them, L. caerulea subsp. altaica, L.caerulea subsp. pallasii, and L. micrantha have been sequenced for the first time to date. We conducted a plastome-based analysis to characterize the plastomes of the selected Lonicera species. Our analysis included comparative plastome assessments with previously sequenced Lonicera species from GenBank, identification of potential molecular markers valuable for DNA barcoding and population genetics, and evaluation of the taxonomic positions of the studied Lonicera species

## Materials and methods

Plant leaf material collection and DNA extraction. Fresh leaf samples were collected from the eastern and central parts of Kazakhstan. Detailed information on the collection sites is provided in Table 1. The leaves were dried in silica gel and subsequently used for DNA extraction. Genomic DNA was extracted from the dried Lonicera leaves using the cetyltrimethylammonium bromide (CTAB) method (Doyle J.J., Doyle J.L., 1987). The quality and concentration of the extracted DNA were assessed using a NanoDrop™ One spectrophotometer (Thermo Fisher Scientific, Waltham, MA, USA).

**Table 1.**
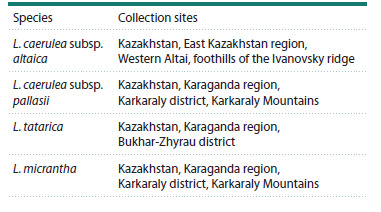
Information on the collection sites of studied Lonicera species in Kazakhstan

Plastid genome sequencing, assembly, and annotation. DNA samples that passed Quality Control (QC) analysis were used for subsequent library preparation. The libraries were constructed using the TruSeq Nano DNA Kit (Illumina Inc., San Diego, CA, USA). Plastid genome sequencing was performed on the Illumina NovaSeq 6000 (Illumina Inc.) platform at Macrogen Inc. (Seoul, Republic of Korea). The quality of raw sequencing data was assessed using FastQC 0.11.7 (http://www.bioinformatics.babraham.ac.uk/projects/fastqc, accessed on 02 December 2024). The adapter sequences were removed from the raw reads using Trimmomatic 0.38 (Bolger et al., 2014). De novo assembly was conducted using NOVOplasty (Dierckxsens et al., 2017), and plastome annotation was performed using the Plastid Genome Annotator (PGA) (Qu et al., 2019). Gene maps of the annotated plastid genomes of L. caerulea subsp. altaica, L.caerulea subsp. pallasii, L. tatarica, and L. micrantha were drawn using the OrganellarGenomeDRAW tool 1.3.1 (OGDRAW) (Lohse et al., 2007). The newly sequenced plastomes of these species have been deposited in GenBank under the accession numbers PV026015-PV026018

Comparative plastome analysis. Comparative plastome analysis of the studied Lonicera species was conducted using mVISTA (Frazer et al., 2004) in Shuffle-LAGAN mode, with the plastid genomes of L. caerulea (OQ784224) and L. tatarica (OQ784187) serving as references. Additionally, the junction sites of the four Lonicera plastomes were examined using the IRscope online tool (Amiryousefi et al., 2018), utilizing the same reference genomes, L. caerulea (OQ784224) and L. tatarica (OQ784187).

Nucleotide variability analysis. The complete plastid genome sequences of the Lonicera species were aligned using Geneious Prime® 2025.0.3 (https://www.geneious.com, accessed on 10 February 2025). The aligned sequences were then analyzed for nucleotide variability (Pi) using a sliding window approach in DnaSP v6 (Rozas et al., 2003). The sliding window analysis was performed with a window length of 600 bp and a step size of 200 bp.

Simple sequence repeats analysis and comparative genome analysis. Simple sequence repeats (SSRs) in the nucleotide sequences of the four studied Lonicera plastomes were identified using MISA software (Beier et al., 2017). The detection thresholds were set as follows: eight repeats for mononucleotide SSRs, four repeats for di- and trinucleotide SSRs, and three repeats for tetra-, penta-, and hexanucleotide SSRs.

Phylogenetic analysis. Phylogenetic analysis was conducted using alignments of complete plastid genome sequences, protein-coding gene sequences, and variable region gene sequences from L. caerulea subsp. altaica, L. caerulea subsp. pallasii, L. tatarica, and L. micrantha, along with GenBank samples, including outgroup species (Heptacodium miconioides and Triosteum himalayanum). A total of 24 complete plastid genomes were selected to construct phylogenetic trees in order to determine the phylogenetic placement of the studied species within the genus Lonicera. The sequence alignment of the complete plastid genomes was conducted using Geneious Prime® 2025.0.3 (https://www.geneious.com, accessed on 12 February 2025). Phylogenetic relationships were inferred using the maximum likelihood (ML) and Bayesian inference (BI) methods. Maximum likelihood trees were generated using IQ-TREE 2.2.2.6 (Nguyen et al., 2015). The software was also used to determine the optimal tree-building model, identified as GTR + F + I + R2 for complete plastid genome and variable region genes data, and as TVM + F + I + R3 for protein-coding genes data, which were then applied to reconstruct the ML phylogenetic tree. BI phylogenetic trees were reconstructed using MrBayes 3.2.7 (Ronquist et al., 2012). The resulting phylogenetic trees were visualized using FigTree (Rambaut, 2009). The network analysis was performed in SplitsTree4 (Huson, Bryant, 2006) with the Neighbor-Net algorithm.

## Results

**General features of the four Lonicera plastomes**

**Fig. 1.**
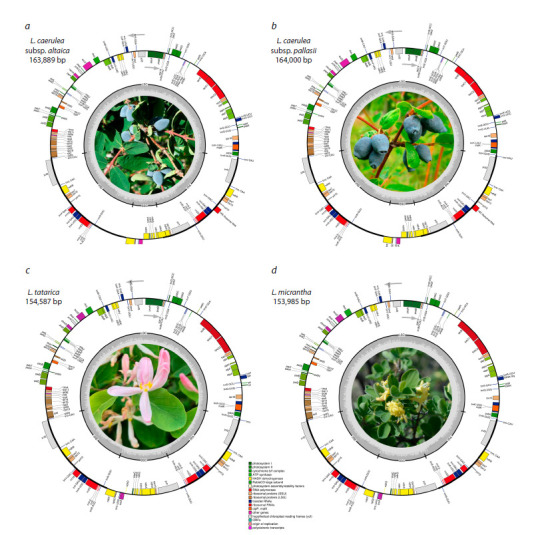
Plastid genome maps of L. caerulea subsp. altaica (a), L. caerulea subsp. pallasii (b), L. tatarica (c) and L. micrantha (d) species Genes positioned outside the outer circle are transcribed in a counterclockwise direction, while those inside the circle are transcribed in a clockwise direction. The inner circle represents GC and AT content, with darker gray indicating GC content and lighter gray representing AT content. Genes are color-coded according to their functional categories. The plastid genome map displays a large single-copy (LSC) region, small single-copy (SSC) region, and inverted repeat regions (IRA and IRB).

Illumina sequencing generated paired-end reads with an average length of 150 bp for the four Lonicera plastomes. The lengths of the plastid genomes of L. caerulea subsp. altaica, L. caerulea subsp. pallasii, L. tatarica, and L. micrantha were 163,889; 164,000; 154,587, and 153,985 bp, respectively (Fig. 1).

The plastid genome structure consisted of a large singlecopy (LSC) region, ranging from 88,040 bp in L. micrantha to 88,813 bp in L. caerulea subsp. pallasii, a small single-copy (SSC) region varying from 10,172 bp in L. caerulea subsp. altaica to 18,750 bp in L. tatarica, and an inverted repeat (IR) region, spanning from 47,356 bp in L. micrantha to 65,598 bp in L. caerulea subsp. altaica. The two inverted repeat regions were designated as IRA and IRB. The total GC content of the four Lonicera plastid genomes was relatively consistent, ranging from 38.05 % in L. caerulea subsp. altaica to 38.42 % in L. tatarica plastome. The IR regions exhibited higher GC content (40.34–43.44 %) compared to the single-copy regions, with the LSC region ranging from 36.83 to 36.95 % and the SSC region from 32.84 to 33.06 % (Table 2).

**Table 2.**
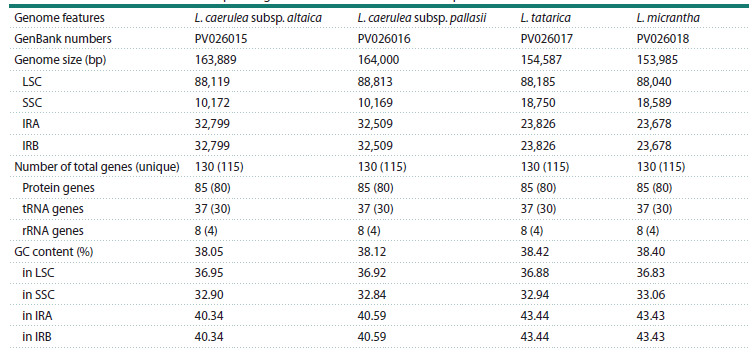
General characteristics of the plastid genomes of the studied Lonicera species

The four assembled plastid genomes of Lonicera exhibited identical gene content, intron numbers, and gene order. The plastid genomes of the studied Lonicera species comprised 130 genes, including 85 protein-coding genes, 37 tRNA genes, and eight rRNA genes. Among them, five protein-coding genes (rps7, rps12, ndhB, ycf2, and ycf15), seven tRNA genes (trnAUGC, trnI-CAU, trnI-GAU, trnL-CAA, trnN-GUU, trnR-ACG, and trnV-GAC), and four rRNA genes (rrn4.5, rrn5, rrn16, and rrn23) were duplicated within the IR regions of the four Lonicera plastid genomes. A total of 17 genes contained introns, of which 16 genes (rps12, rps16, rpl2, rpl16, rpoC1, atpF, ndhA, ndhB, petB, petD, trnA-UGC, trnG-UCC, trnIGAU, trnK-UUU, trnL-UAA, and trnV-UAC) had a single intron, while ycf3 was the only gene containing two introns. The rps12 gene exhibited trans-splicing, with its 5′ end located in the LSC region, while its 3′ end was positioned in the IR regions (Fig. 1, Table 3).

**Table 3.**
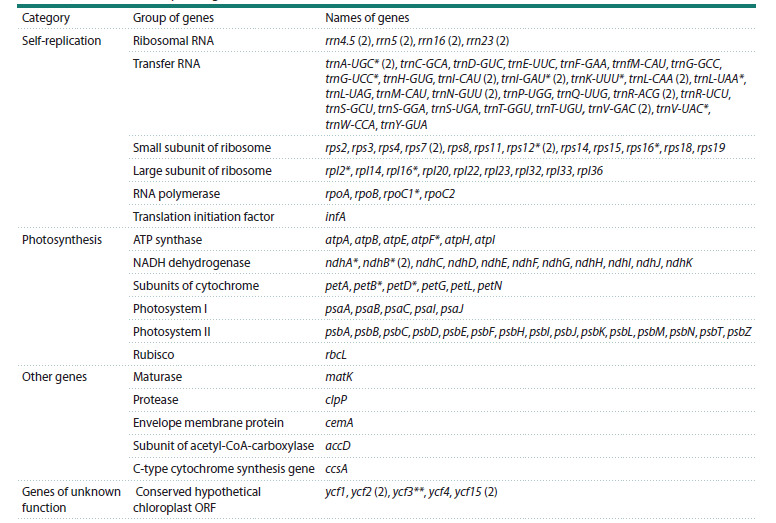
Gene composition and functional categorization of the L. caerulea subsp. altaica, L. caerulea subsp. pallasii, L. tatarica, and L. micrantha plastid genomes * Genes containing a single intron, genes containing two introns; (2) – duplicated genes.

**Comparative analysis of the four Lonicera plastomes**

**Fig. 2.**
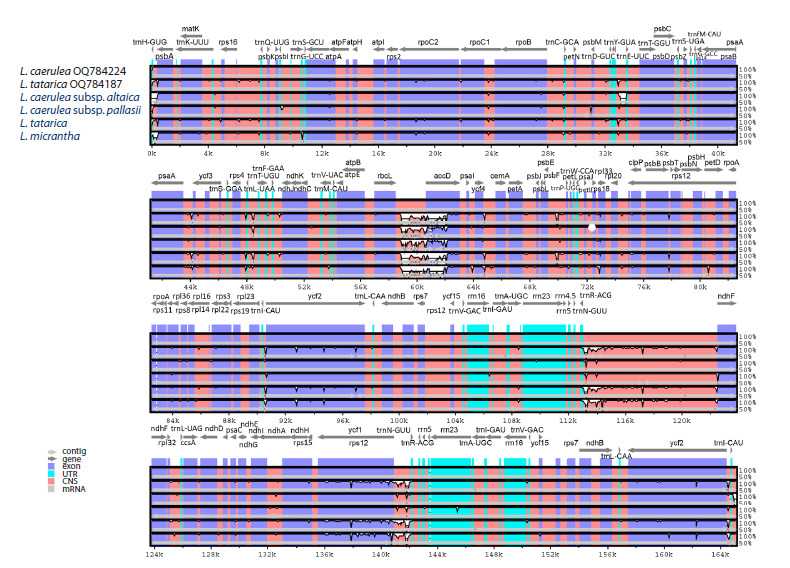
Comparison of complete plastid genomes of six Lonicera samples using mVISTA, with L. caerulea (OQ784224) and L. tatarica (OQ784187) as reference genomes Gray arrows above the alignment indicate gene locations, while different colors distinguish coding and non-coding regions. The horizontal axis represents plastome coordinates, and the vertical scale depicts sequence identity percentages, ranging from 50 to 100 %.

A comparative analysis of the complete plastid genomes of six Lonicera species was conducted using mVISTA, with L. caerulea (OQ784224) and L. tatarica (OQ784187) as reference genomes. The alignment revealed high sequence conservation across the plastomes, with most variations occurring in noncoding regions. Among the coding regions, accD exhibited the highest level of divergence. The IR regions were the most conserved, while the LSC and SSC regions displayed higher levels of sequence divergence (Fig. 2).

**Inverted repeat expansion and contraction**

**Fig. 3.**
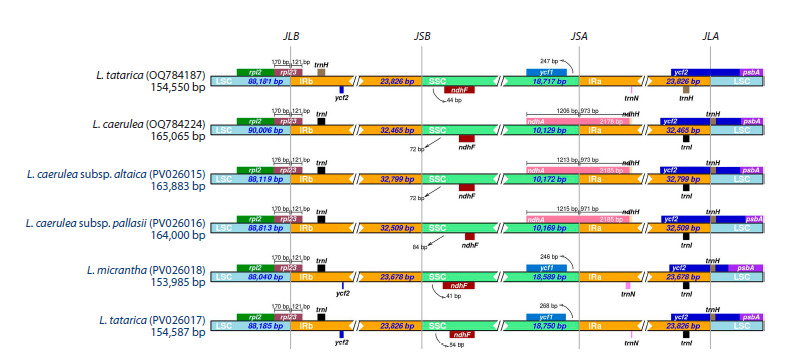
Comparison of the junctions between the LSC, IR, and SSC regions in Lonicera plastomes. Species highlighted in blue were analyzed in this study. JLB represents the junction between the LSC and IRB regions, JSB marks the boundary between the IRB and SSC regions; JSA indicates the junction between the SSC and IRA regions, and JLA denotes the boundary between the IRA and LSC regions.

A comparative analysis of the LSC/IRB/SSC/IRA boundary regions was conducted in the plastomes of Lonicera species (L. caerulea subsp. altaica, L. caerulea subsp. pallasii, L. tatarica, and L. micrantha), using L. caerulea (OQ784224) and L. tatarica (OQ784187) from GenBank as reference sequences. There were structural differences in LSC/IRB/SSC/ IRA boundaries of Lonicera plastomes. The length of the IR regions ranged from 23,678 to 32,799 bp in four studied Lonicera plastomes with some expansion. A notable difference was found in L. caerulea subsp. altaica and L. caerulea subsp. pallasii plastomes, where the gene ndhA, which crossed over the IRA/SSC boundaries, was similar to those in GenBank (L. caerulea). The ycf1 gene’s distance from the IRA region was 246 and 268 bp in L. tatarica, and L. micrantha, respectively. At the IRB/SSC border, the ndhF gene was fully present within the SSC region in all Lonicera plastomes, extending into the IRB region with lengths ranging from 41 to 84 bp (Fig. 3).

**Nucleotide diversity analysis**

**Fig. 4.**
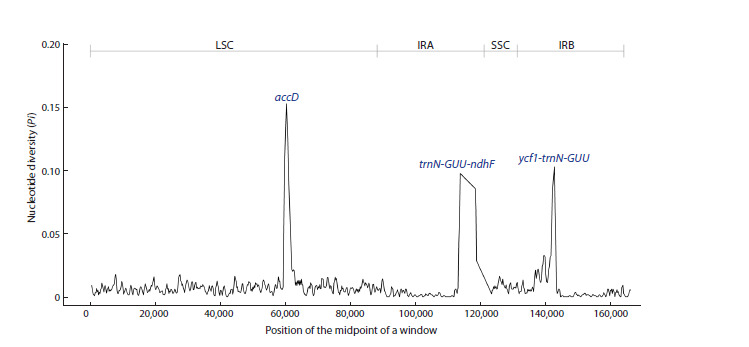
Nucleotide diversity of the four Lonicera plastid genomes using sliding window analysis (window length – 600 bp, step size – 200 bp). The X-axis represents the midpoint position of each window, while the Y-axis denotes the nucleotide diversity (Pi) value for each window.

To assess nucleotide diversity values, the four Lonicera complete plastid genomes in this study were aligned. The aligned nucleotide sequences were then analyzed to calculate the nucleotide diversity of the plastid genome using DnaSP. The results revealed that the Pi values in the four Lonicera plastomes ranged from 0 to 0.15222. Three highly variable regions were identified: two intergenic regions ( ycf1-trnNGUU and trnN-GUU-ndhF) and one genic region (accD). Among these, the accD gene region exhibited the highest Pi value (0.15222), followed by the ycf1-trnN-GUU region (0.10250) and trnN-GUU-ndhF (0.09722). Notably, the accD region with the highest nucleotide diversity was concentrated in the LSC region of the plastid genome (Fig. 4).

**Repeat sequence analysis**

This study identified 163, 163, 158, and 157 SSRs in the plastid genomes of Lonicera caerulea subsp. altaica, L. caerulea subsp. pallasii, L. tatarica, and L. micrantha, respectively, resulting in a total of 641 SSRs. Five types of SSRs were identified, including mono-, di-, tri-, tetra-, and hexa-nucleotide repeats. Most of the identified SSR markers were located within the intergenic regions of the plastid genome’s LSC region. Detailed information is provided in Supplementary Table S11. Mononucleotide repeats were the most abundant SSR motifs, comprising approximately 72.70 % of the total SSRs, followed by dinucleotide repeats (18.72 %) and tetranucleotide repeats (5.93 %). The most abundant SSR motifs were mononucleotide repeats, which accounted for approximately 72.70 % of the total SSRs, followed by dinucleotide (18.72 %) and tetranucleotide (5.93 %) repeats. Most of the mononucleotide repeats consisted of A/T (451) rather than C/G (15), while the majority of dinucleotide repeats were composed of AT/AT (75) rather than AG/CT (45). Trinucleotide (1.25 %) and hexanucleotide (1.40 %) repeats were rare across the studied plastid genomes but were present in all four plastomes. Pentanucleotide repeats were not found in any of the studied plastomes (Table 4).


Supplementary Materials are available in the online version of the paper: https://vavilovj-icg.ru/download/pict-2025-29/appx29.xlsx


**Table 4.**
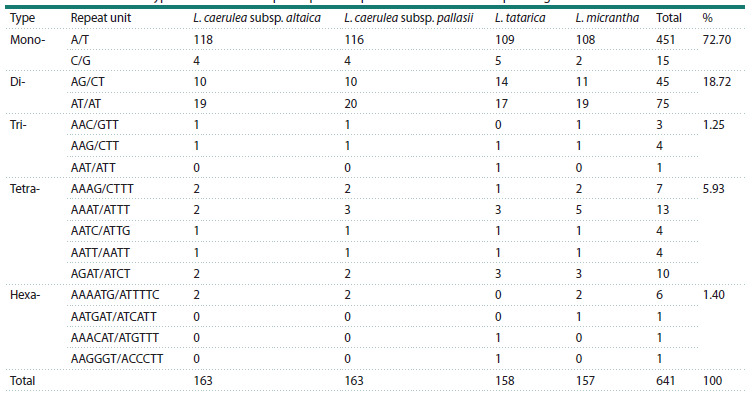
The number and types of identified simple sequence repeats in the four Lonicera plastid genomes

**Phylogenetic analysis**

**Fig. 5.**
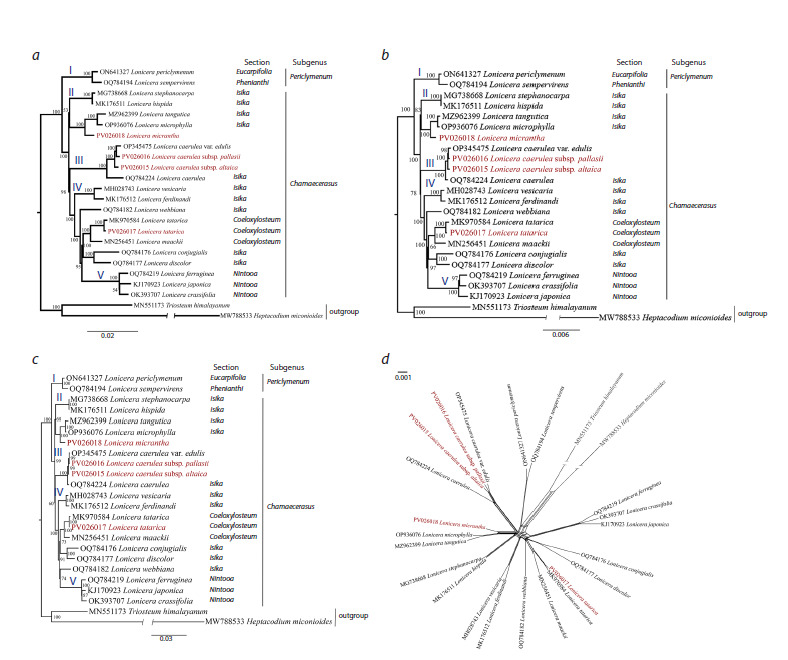
Maximum likelihood phylogenetic tree of the genus Lonicera inferred from nucleotide sequences of the complete plastid genome (a), proteincoding genes (b), and variable region genes (c). Numbers at the nodes of the phylogenetic trees represent bootstrap support values Roman numerals (I–V) at the nodes of the phylogenetic trees denote subclade numbers. Splitstree neighbour-net network of 22 Lonicera and 2 outgroup plastid genomes (d). Species highlighted in red were sequenced in this study.

The ML method was used to reconstruct the phylogenetic trees based on nucleotide sequences of complete plastid genomes (Fig. 5a), protein-coding genes (Fig. 5b), and variable region genes (Fig. 5c). H. miconioides and T. himalayanum were used as outgroups, while 22 Lonicera samples were included as ingroups. The ML trees showed that the analyzed Lonicera species were grouped into two clades: the Periclymenum subgenus clade and the Chamaecerasus subgenus clade. There were five subclades in the ML phylogenetic trees (Fig. 5a–c), which represented the sections Eucarpifolia and Phenianthi (Subclade I) within subgenus Periclymenum, section Isika (Subclades II and III), sections Isika and Coeloxylosteum (Subclades IV), and section Nintooa (Subclade V) within subgenus Chamaecerasus. The phylogenetic tree reveals that L. tatarica forms a subclade (IV) consisting of species from the Coeloxylosteum section, clustering with the L. tatarica (MK970584) sequence from GenBank and indicating a close relationship with L. maackii (MN256451) from GenBank. Furthermore, L. caerulea subsp. altaica and L. caerulea subsp. pallasii samples analyzed in this study clustered in one subclade (III) with the L. caerulea (OQ784224) and L. caerulea subsp. edulis (OP345475) sequences from GenBank. Also, L. micrantha is positioned within the Chamaecerasus subgenus in subclade II and clusters closely with species from the Isika section, particularly L. tangutica (MZ962399) and L. microphylla (OP936076) from GenBank. Most of the described subclades exhibit strong bootstrap support (100 %) at the corresponding nodes, except for subclade II in the ML phylogenetic tree based on complete plastid genome data (Fig. 5a), which has moderate support (53 %), indicating high confidence in their overall phylogenetic relationships.

The BI phylogenetic trees were constructed using the same set of samples based on nucleotide sequences from complete plastid genomes (Fig. S1), protein-coding genes (Fig. S2), and variable region genes (Fig. S3). The resulting trees consistently divided the analyzed Lonicera species into two distinct clades corresponding to the subgenera Periclymenum and Chamaecerasus. The topologies of the BI phylogenetic trees were largely congruent with those obtained using ML methods.

To further investigate the relationships and potential reticulation within Lonicera species, we constructed a SplitsTree phylogenetic network (Fig. 5d) based on complete plastid genome sequences from 22 Lonicera and two outgroup samples.

In this analysis, relationships were generally congruent with those in ML and BI phylogenetic trees. The results of the phylogenetic network coincided with the ML dendrogram (Fig. 5a–c). The network indicates that L. micrantha has evolved significantly earlier than L. microphylla and L. tangutica. Also, three subspecies of L. caerulea (altaica, pallasii, and edulis) seem to be hybrid forms of the species (Fig. 5d).

## Discussion

In the present study, the complete plastid genomes of the L. caerulea subsp. altaica, L. caerulea subsp. pallasii, L. tatarica, and L. micrantha were sequenced using next-generation sequencing technology. These genomes were then compared with those of other Lonicera species to enhance our understanding of the molecular taxonomy of the genus.

The plastid genomes of the studied Lonicera exhibited the typical circular structure found in angiosperms (Palmer et al., 1988; Ruhlman, Jansen, 2014), consisting of an LSC region, an SSC region, and two IR regions (Fig. 1). Our annotation identified a total of 130 genes, including 115 unique genes, consisting of 85 (80 unique) protein-coding genes, 37 (30 unique) tRNA genes, and eight (4 unique) rRNA genes (Table 3). Previous studies have reported slightly different numbers of annotated protein-coding genes, with 82 and 83 genes identified in earlier analyses (He et al., 2017; Liu M.L. et al., 2018; Yang C. et al., 2023). The discrepancies in gene annotation primarily arise from differences in the ycf15 gene, which are lost in L. japonica, L. ferdinandi, L. hispida, L. nervosa, L. fragrantissima var. lancifolia, L. stephanocarpa, L. tragophylla, L. acuminata, and L. similis plastomes. Additionally, we identified the trnM-CAU gene, which was not annotated in previous Lonicera plastome studies (Frazer et al., 2004). These variations underscore the importance of annotation accuracy, indicating that further comparative analyses are necessary to refine gene identification within the genus.

The genome sizes varied among species, ranging from 153,985 bp in L. micrantha to 164,000 bp in L. caerulea subsp. pallasii. Notably, the plastid genome sizes of L. caerulea subsp. altaica (163,889 bp) and L. caerulea subsp. pallasii (164,000 bp) were larger than those of the other two (L. tatarica, and L. micrantha) studied species (Table 2). The variations in the total length of plastid genomes are typically associated with the expansion and contraction of IR regions (Zhang X.F. et al., 2021). In this study, the IR regions of L. caerulea subsp. altaica (32,799 bp) and L. caerulea subsp. pallasii (32,509 bp) were found to be longer than the SSC and LSC regions, contributing to their relatively larger plastome sizes (Fig. 3). These results align with the previously reported plastid genome size of L. caerulea (165,065 bp) (Yang X.L. et al., 2024), suggesting that the specific plastid genome lengths observed in the studied L. caerulea subspecies may be a common evolutionary characteristic with the L. caerulea species

Molecular markers are essential tools in modern biological research, playing a crucial role in unraveling genetic diversity, phylogenetic relationships, and population dynamics (Wang X.R., Szmidt, 2001; Al-Hadeithi, Jasim, 2021). Among them, DNA barcoding markers offer an efficient approach to species identification by targeting short, conserved regions of the genome (Chac, Thinh, 2023). These markers have made one of the most significant contributions to advancing our understanding of evolutionary processes, establishing DNA barcoding as a core methodology in plant taxonomy (Purty, Chatterjee, 2016; Zhu S. et al., 2022). The highly variable regions in nucleotide sequences of the plastid genome can be used as potential specific DNA barcoding markers for specific plant genera. Using mVISTA (Fig. 2) and sliding window analysis (Fig. 4), we identified three highly variable regions in this study: two intergenic regions (ycf1-trnN-GUU and trnN-GUU-ndhF) and one genic region (accD). These regions show promise as DNA barcoding markers for the phylogenetic analysis of Lonicera species. Notably, the trnN-GUU-ndhF region has been reported as particularly useful for developing molecular markers in Lonicera species (Liu M.L. et al., 2018; Yang C. et al., 2023).

Our study found that the accD gene region is the most variable, a finding consistent with previous studies in Asteraceae (Kim et al., 2020) and Fabaceae (Zhang T. et al., 2024). Two intergenic regions (ycf1-trnN-GUU and trnN-GUU-ndhF) identified in this study were also reported in other plant species. For example, ycf1-trnN-GUU was a highly variable region in the plastid genomes of Parasenecio (Liu X. et al., 2023) and Medicago (Jiao et al., 2023) species. Z. Cao et al. (2023) and W. Xing et al. (2024) reported that the trnN- GUU- ndhF intergenic region is hypervariable in the plastid genomes of Neocinnamomum taxa and Pinellia ternata, respectively. This study identified two highly variable intergenic regions and one genic region as promising candidates for DNA barcoding markers in future research. Nonetheless, further studies are needed to assess the effectiveness of these divergent markers in the phylogenetic analysis of Lonicera species.

Another important class of molecular markers is the simple sequence repeat (SSR) markers, which are widely recognized for their value in plant population genetics in assessing genetic diversity, population structure, and evolutionary relationships (Chen F. et al., 2015; Yermagambetova et al., 2024). In our study, we identified a total of 641 SSR markers across the plastid genomes analyzed, with individual counts ranging from 158 in L. tatarica to 163 in both L. caerulea subsp. altaica and L. caerulea subsp. pallasii plastid genomes (Table 4). Notably, the majority of these SSRs were located in the intergenic regions of the LSC region, a distribution pattern that aligns well with previous findings on angiosperm plastomes (Xia C. et al., 2022; Nyamgerel et al., 2024).

Our results reveal that mononucleotide repeats are the most prevalent SSR motifs across the four Lonicera plastomes analyzed, which are common for Caprifoliaceae representatives (Liu H. et al., 2022; Wang L. et al., 2024). Notably, the majority of the mononucleotide repeats were composed of A/T (451) rather than C/G (15), while dinucleotide repeats were predominantly composed of AT/AT (75) as opposed to AG/CT (45). This distribution is consistent with patterns observed in plastid genomes of many other plant species (Li X.Q. et al., 2019; Souza et al., 2019). Numerous studies have demonstrated that SSR markers derived from plastid genome sequences are effective for assessing genetic diversity in plant populations (Jo et al., 2022; Lācis et al., 2022; Guo et al., 2025). The SSR markers identified in our study hold potential for population genetic analyses within the genus Lonicera. However, further validation is required to confirm their efficacy and reliability in elucidating the genetic structure of Lonicera species populations.

Plastid genome nucleotide sequences have become a powerful tool in phylogenetic studies of different plant genera (Wu et al., 2021; Xia Q. et al., 2023). Their conserved structure, uniparental inheritance, and relatively slow mutation rate make them ideal for resolving evolutionary relationships across diverse plant lineages (Chen J. et al., 2022; Feng et al., 2024). With the development of next-generation sequencing technologies, the rapid and cost-effective sequencing of entire plastid genomes has become increasingly accessible, enhancing their utility in plant taxonomy by providing greater phylogenetic resolution and a deeper understanding of plant evolutionary history. This study utilized complete plastome sequences, protein-coding gene sequences, and variable region gene sequences for the phylogenetic analysis of the studied Lonicera species (L. caerulea subsp. altaica, L. caerulea subsp. pallasii, L. tatarica, and L. micrantha), along with publicly available sequences from GenBank, to contribute to a better understanding of phylogenetic relationships within the genus. The ML trees based on the sequences of the complete plastid genome (Fig. 5a), protein-coding genes (Fig. 5b), and variable region genes (Fig. 5c) of the 22 Lonicera samples and two outrgoup samples (H. miconioides and T. himalayanum) was reconstructed.

The phylogenetic analyses in this study revealed that the Lonicera species were grouped into two major clades, corresponding to the subgenera Periclymenum and Chamaecerasus, which is consistent with previous phylogenetic studies (Srivastav et al., 2023; Yang X.L. et al., 2024). Furthermore, the larger clade representing subgenus Chamaecerasus was further divided into four distinct subclades corresponding to sections Isika (Subclades II and III), sections Isika and Coeloxylosteum (Subclades IV), and section Nintooa (Subclade V). Within subgenus Chamaecerasus, species are subclustering into four subclades and align with recognized sectional classifications (Srivastav et al., 2023).

The placement of L. micrantha, L. caerulea subsp. altaica and L. caerulea subsp. pallasii, which had not been previously assigned to a section, suggests their belonging to the Isika section based on their close clustering with other members of this section. Additionally, the finding supports the assumption that L. caerulea subsp. altaica and L. caerulea subsp. pallasii share a common evolutionary history with L. caerulea, which is also supported by the plastid genome structure of these species. Furthermore, L. tatarica forms a subclade (IV) with L. tatarica (MK970584) and L. maackii (MN256451) from GenBank, grouping within the Coeloxylosteum section. Notably, these samples are positioned between the species of section Isika, suggesting a possible evolutionary relationship between these two sections

The phylogenetic analysis aimed to clarify the phylogenetic positions of the studied Lonicera species from Kazakhstan using plastid genome nucleotide sequences, including three newly sequenced ones (L. caerulea subsp. altaica, L. caerulea subsp. pallasii, and L. micrantha) in this study. The genomic data obtained in this study provide valuable resources for future phylogenetic research, contributing to an understanding of evolutionary relationships within the genus Lonicera and supporting further taxonomic revisions. Based on the comparison of phylogenetic trees reconstructed using different datasets and methods, we conclude that the maximum likelihood tree derived from complete plastid genome sequences was the most informative, and its topology is consistent with those reported in previous studies (Srivastav et al., 2023; Yang X.L. et al., 2024). Furthermore, the nucleotide sequences of variable regions such as accD-ycf1-ndhF-trnN-GUU also demonstrate high potential for use in DNA barcoding, and may serve as valuable molecular markers for species phylogenetic studies within the genus Lonicera

## Conclusion

The complete plastid genomes of L. caerulea subsp. altaica, L. caerulea subsp. pallasii, L. tatarica, and L. micrantha exhibited the typical circular structure with four distinct regions. Structural variations were observed in the plastomes of L. caerulea subsp. altaica and L. caerulea subsp. pallasii, particularly in genome sizes, which were larger than in the other two species (L. tatarica and L. micrantha) due to an extended IR region. This finding aligns with previous studies on L. caerulea plastomes, further supporting their shared evolutionary history. The nucleotide sequences of variable regions such as accD-ycf1-ndhF-trnN-GUU demonstrate high potential for use in DNA barcoding, and may serve as valuable molecular markers for species phylogenetic studies within the genus Lonicera. Further studies are required to assess the effectiveness of the identified simple sequence repeats.

## Conflict of interest

The authors declare no conflict of interest.

## References

Abdulina S.A. Checklist of Vascular Plants of Kazakhstan. Almaty,
1999 (in Russian)

Al-Hadeithi Z.S.M., Jasim S.A. Study of plant genetic variation through
molecular markers: an overview. J Pharm Res Int. 2021;33:464-473.
doi 10.9734/jpri/2021/v33i45B32828

Ali I., Khan D., Ali F., Bibi H., Malik A. Phytochemical, antioxidant
and antifungal studies on the constituents of Lonicera quinquelocularis.
J Chem Soc Pak. 2013;35(1):139-143. doi 10.5897/JMPR
2013.5245

Almerekova S., Yermagambetova M., Ivashchenko A., Abugalieva S.,
Turuspekov Y. Assessment of complete plastid genome sequences of
Tulipa alberti Regel and Tulipa greigii Regel species from Kazakhstan.
Genes. 2024;15:1447. doi 10.3390/genes15111447

Ametov A.A., Mukhitdinov N.M., Abidkulova K.T., Karasholakova
L.N., Ydyrys A. Characteristics of plant communities with
Lonicera iliensis Pojark. in the middle flow of the River Ili. KazNU
Bulletin. Biol Series. 2016;4(69):12-21 (in Russian)

Amiryousefi A., Hyvönen J., Poczai P. IRscope: an online program to
visualize the junction sites of chloroplast genomes. Bioinformatics.
2018;34:3030-3031. doi 10.1093/bioinformatics/bty220

Baitulin I.O., Sitpayeva G.T. (Eds) Red Book of Kazakhstan: Plants.
Astana, 2014 (in Russian)

Beier S., Thiel T., Munch T., Scholz U., Mascher M. MISA-web: a web
server for microsatellite prediction. Bioinformatics. 2017;33(16):
2583-2585. doi 10.1093/bioinformatics/btx198

Bolger A.M., Lohse M., Usadel B. Trimmomatic: a flexible trimmer
for Illumina sequence data. Bioinformatics. 2014;30(15):2114-2120.
doi 10.1093/bioinformatics/btu170

Boyarskikh I.G., Kostikova V.A. Changes in the individual and group
composition of polyphenols in leaves of Lonicera caerulea subsp.
altaica and Spiraea chamaedryfolia as related to chemical element
content in soil and plants on ultra-alkaline parent rock material.
Rastitelnye Resursy. 2023;59(2):164-179. doi 10.31857/S0033
994623020048 (in Russian)

Cao Z., Yang L., Xin Y., Xu W., Li Q., Zhang H., Tu Y., Song Y., Xin P.
Comparative and phylogenetic analysis of complete chloroplast genomes
from seven Neocinnamomum taxa (Lauraceae). Front Plant
Sci. 2023;14:1205051. doi 10.3389/fpls.2023.1205051

Chac L.D., Thinh B.B. Species identification through DNA barcoding
and its applications: a review. Biol Bull. 2023;50:1143-1156. doi
10.1134/s106235902360229x

Chen F., Liu H., Yao Q., Fang P., Lv F. Genetic variations and evolutionary
relationships among radishes (Raphanus sativus L.) with
different flesh colors based on red pigment content, karyotype and
simple sequence repeat analysis. Afr J Biotechnol. 2015;16:3270-
3281. doi 10.5897/AJB2015.14911

Chen J., Xie D., He X., Yang Y., Li X. Comparative analysis of the complete
chloroplast genomes in Allium section Bromatorrhiza species
(Amaryllidaceae): phylogenetic relationship and adaptive evolution.
Genes. 2022;13(7):1279. doi 10.3390/genes13071279

Chen X., Zhou J., Cui Y., Wang Y., Duan B., Yao H. Identification of
Ligularia herbs using the complete chloroplast genome as a superbarcode.
Front Pharmacol. 2018;9:695. doi 10.3389/fphar.2018.
00695

Dierckxsens N., Mardulyn P., Smits G. NOVOPlasty: de novo assembly
of organelle genomes from whole genome data. Nucleic Acids Res.
2017;45(4):e18. doi 10.1093/nar/gkw955

Dong S., Ying Z., Yu S., Wang Q., Liao G., Ge Y., Cheng R. Complete
chloroplast genome of Stephania tetrandra (Menispermaceae) from
Zhejiang Province: insights into molecular structures, comparative
genome analysis, mutational hotspots and phylogenetic relationships.
BMC Genomics. 2021;22(1):880. doi 10.1186/s12864-021-08193-x

Donoghue M.J., Bell C.D., Li J. Phylogenetic patterns in Northern
Hemisphere plant geography. Int J Plant Sci. 2001;162(S6):S41-
S52. doi 10.1086/323278

Doyle J.J., Doyle J.L. A rapid DNA isolation procedure for small quantities
of fresh leaf tissue. Phytochem Bull. 1987;19(1):11-15

Feng Z., Zheng Y., Jiang Y., Pei J., Huang L. Phylogenetic relationships,
selective pressure and molecular markers development of six species
in subfamily Polygonoideae based on complete chloroplast genomes.
Sci Rep. 2024;14(1):9783. doi 10.1038/s41598-024-58934-7

Frazer K.A., Pachter L., Poliakov A., Rubin E.M., Dubchak I. VISTA:
computational tools for comparative genomics. Nucleic Acids Res.
2004;32:W273-W279. doi 10.1093/nar/gkh458

Ge L., Li J., Wan H., Zhang K., Wu W., Zou X., Wu S., Zhou B., Tian J.,
Zeng X. Novel flavonoids from Lonicera japonica flower buds and
validation of their anti-hepatoma and hepatoprotective activity
in vitro studies. Ind Crops Prod. 2018;125:114-122. doi 10.1016/
j.indcrop.2018.08.073

Golubev D., Zemskaya N., Shevchenko O., Shaposhnikov M., Kukuman
D., Patov S., Punegov V., Moskalev A. Honeysuckle extract
(Lonicera pallasii L.) exerts antioxidant properties and extends the
lifespan and healthspan of Drosophila melanogaster. Biogerontology.
2022;23(2):215-235. doi 10.1007/s10522-022-09954-1

Guo Q., Xue X., Wang D., Zhang L., Liu W., Wang E., Cui X., Hou X.
Genetic diversity and population genetic structure of Paeonia suffruticosa
by chloroplast DNA simple sequence repeats (CpSSRs).
Hortic Plant J. 2025;11(1):367-376. doi 10.1016/j.hpj.2023.10.006

Hara H. A revision of Caprifoliaceae of Japan with Reference to Allied
Plants in other Districts and the Adoxaceae. Ginkgoana. Tokyo:
Academia Scientific Book, 1983

Hayes D.J., Peterson B.J. Growth of Lonicera caerulea across fertility
and moisture conditions: comparisons with Lonicera villosa and invasive
congeners. HortScience. 2020;55(2):149-155. doi 10.21273/
HORTSCI14318-19

He L., Qian J., Li X., Sun Z., Xu X., Chen S. Complete chloroplast
genome of medicinal plant Lonicera japonica: genome rearrangement,
intron gain and loss, and implications for phylogenetic studies.
Molecules. 2017;22(2):249. doi 10.3390/molecules22020249

Hong Z., He W., Liu X., Tembrock L.R., Wu Z., Xu D. Comparative
analyses of 35 complete chloroplast genomes from the genus Dalbergia
(Fabaceae) and the identification of DNA barcodes for tracking
illegal logging and counterfeit rosewood. Forests. 2022;13(4):
626. doi 10.3390/f13040626

Howe C.J., Barbrook A.C., Koumandou V.L., Nisbet R.E.R., Symington
H.A., Wightman T.F. Evolution of the chloroplast genome.
Philos Trans R Soc Lond B Biol Sci. 2003;358(1429):99-106. doi
10.1098/rstb.2002.1176

Hsu P.S., Hu C.C., Wang H.J. Flora Reipublicae Popularis Sinicae.
Vol. 72. Science Press, 1988 (in Chinese)Huson D.H., Bryant D. Application of phylogenetic networks in evolutionary
studies. Mol Biol Evol. 2006;23(2):254-267. doi 10.1093/
molbev/msj030

Jiao Y.X., He X.F., Song R., Wang X.M., Zhang H., Aili R., Chao Y.H.,
Shen Y.H., Yu L.X., Zhang T.J., Jia S.G. Recent structural variations
in the Medicago chloroplast genomes and their horizontal transfer
into nuclear chromosomes. J Syst Evol. 2023;61(4):627-642. doi
10.1111/jse.12900

Jo I.H., Han S., Shim D., Ryu H., Hyun T.K., Lee Y. Complete chloroplast
genome of the inverted repeat-lacking species Vicia bungei and
development of polymorphic simple sequence repeat markers. Front
Plant Sci. 2022;13:891783. doi 10.3389/fpls.2022.891783

Kim G.B., Lim C.E., Kim J.S., Kim K., Lee J.H., Yu H.J., Mun J.H.
Comparative chloroplast genome analysis of Artemisia (Asteraceae)
in East Asia: insights into evolutionary divergence and phylogenomic
implications. BMC Genomics. 2020;21(1):415. doi 10.1186/
s12864-020-06812-7

Kushnarenko S.V., Karasholakova L.N., Ozek G., Abidkulova K.T.,
Mukhitdinov N.M., Baser K.H.C., Ozek T. Investigation of essential
oils from three natural populations of Lonicera iliensis. Chem Nat
Compd. 2016;52:751-753. doi 10.1007/s10600-016-1765-6

Li Q. The complete chloroplast genomes of Primula obconica provide
insight that neither species nor natural section represent monophyletic
taxa in Primula (Primulaceae). Genes. 2022;13(4):567. doi
10.3390/genes13040567

Li X.Q., Zuo Y.J., Zhu X.X., Liao S., Ma J.S. Complete chloroplast
genomes and comparative analysis of sequence evolution among
seven Aristolochia (Aristolochiaceae) medicinal species. Int J Mol
Sci. 2019;20(5):1045. doi 10.3390/ijms20051045

Lin L.M., Zhang X.G., Zhu J.J., Gao H.M., Wang Z.M., Wang W.H.
Two new triterpenoid saponins from the flowers and buds of
Lonicera japonica. J Asian Nat Prod Res. 2008;10(10):925-929. doi
10.1080/10286020802217366

Liu H., Liu W., Ahmad I., Xiao Q., Li X., Zhang D., Fang J., Zhang G.,
Xu B., Gao Q., Chen S. Complete chloroplast genome sequence of
Triosteum sinuatum, insights into comparative chloroplast genomics,
divergence time estimation and phylogenetic relationships among
Dipsacales. Genes. 2022;13(5):933. doi 10.3390/genes13050933

Liu M.L., Fan W.B., Wang N., Dong P.B., Zhang T.T., Yue M., Li Z.H.
Evolutionary analysis of plastid genomes of seven Lonicera L. species:
implications for sequence divergence and phylogenetic relationships.
Int J Mol Sci. 2018;19(12):4039. doi 10.3390/ijms19124039

Liu M., Yu Q., Yi Y., Xiao H., Putra D.F., Ke K., Zhang Q., Li P. Antiviral
activities of Lonicera japonica Thunb. components against
grouper iridovirus in vitro and in vivo. Aquaculture. 2020;519:
734882. doi 10.1016/j.aquaculture.2019.734882

Liu X., Luo J., Zhang M., Wang Q., Liu J., Wu D., Fu Z. Phylogenomic
analysis of two species of Parasenecio and comparative analysis
within tribe Senecioneae (Asteraceae). Diversity. 2023;15:563. doi
10.3390/d15040563

Lohse M., Drechsel O., Bock R. OrganellarGenomeDRAW (OGDRAW):
a tool for the easy generation of high-quality custom graphical maps
of plastid and mitochondrial genomes. Curr Genet. 2007;52:267-
274. doi 10.1007/s00294-007-0161-y

Luo C., Huang W., Sun H., Yer H., Li X., Li Y., Yan B., Wang Q., Wen Y.,
Huang M., Huang H. Comparative chloroplast genome analysis of
Impatiens species (Balsaminaceae) in the Karst area of China: insights
into genome evolution and phylogenomic implications. BMC
Genomics. 2021;22(1):571. doi 10.1186/s12864-021-07807-8

Lācis G., Kārkliņa K., Bartulsons T., Stalažs A., Jundzis M., Baļķe I.,
Ruņģis D., Strautiņa S. Genetic structure of a Ribes genetic resource
collection: inter- and intra-specific diversity revealed by chloroplast
DNA simple sequence repeats (CpSSRs). Sci Hortic. 2022;304:
111285. doi 10.1016/j.scienta.2022.111285

Maximowicz C.J. Diagnoses Plantarum Novarum Asiaticarum. Petropoli,
Imperialis Academiae Scientiarum, 1877. doi 10.5962/bhl.title.
46308

Nakai T. A new classification of the genus Lonicera in the Japanese
Empire, together with the diagnoses of new species and new
varieties.
J Jpn Bot. 1938;14:359-375

Nakaji M., Tanaka N., Sugawara T. A molecular phylogenetic study
of Lonicera L. (Caprifoliaceae) in Japan based on chloroplast DNA
sequences. Acta Phytotaxon Geobot. 2015;66(3):137-151. doi
10.18942/apg.KJ00010115701

Nguyen L.T., Schmidt H.A., Von Haeseler A., Minh B.Q. IQ-TREE:
a fast and effective stochastic algorithm for estimating maximumlikelihood
phylogenies. Mol Biol Evol. 2015;32:268-274. doi 10.1093/
molbev/msu300

Ni F.Y. Chemical constituents from flower buds of Lonicera japonica.
Chin Tradit Herb Drugs. 2017;48(18):3689-3692. doi 10.7501/
j.issn.0253-2670.2017.18.004

Nyamgerel N., Baasanmunkh S., Oyuntsetseg B., Tsegmed Z., Bayarmaa
G., Lazkov G., Pyak E., Gil H.Y., Park I., Choi H.J. Comparative
plastome analysis and taxonomic classification of snow lotus species
(Saussurea, Asteraceae) in Central Asia and Southern Siberia. Funct
Integr Genomics. 2024;24(2):42. doi 10.1007/s10142-024-01309-y

Oyuntsetseg D., Nyamgerel N., Baasanmunkh S., Oyuntsetseg B., Urga-mal
M., Yoon J.W., Bayarmaa G.A., Choi H.J. The complete chloroplast
genome and phylogenetic results support the species position of
Swertia banzragczii and Swertia marginata (Gentianaceae) in Mongolia.
Bot Stud. 2024;65(1):11. doi 10.1186/s40529-024-00417-z

Palmer J.D., Jansen R.K., Michaels H.J., Chase M.W., Manhart J.R.
Chloroplast DNA variation and plant phylogeny. Ann Missouri Bot
Gard. 1988;75(4):1180-1206. doi 10.2307/2399279

Park H.S., Park K.I., Lee D.H., Kang S.R., Nagappan A., Kim J.A.,
Kim E.H., Lee W.S., Shin S.C., Hah Y.S., Kim G.S. Polyphenolic extract
isolated from Korean Lonicera japonica Thunb. induces G2/M
cell cycle arrest and apoptosis in HepG2 cells: involvement of PI3K/
Akt and MAPKs. Food Chem Toxicol. 2012;50(7):2407-2416. doi
10.1016/j.fct.2012.04.034

Purty R.S., Chatterjee S. DNA barcoding: an effective technique in molecular
taxonomy. Austin J Biotechnol Bioeng. 2016;3(1):1059

Qu X.J., Moore M.J., Li D.Z., Yi T.S. PGA: a software package for
rapid, accurate, and flexible batch annotation of plastomes. Plant
Methods. 2019;15:50. doi 10.1186/s13007-019-0435-7

Rambaut A. FigTree, a graphical viewer of phylogenetic trees. 2009.
Available: http://tree.bio.ed.ac.uk/software/figtree/

Rehder A. Synopsis of the genus Lonicera. Mo Bot Gard Annu Rep.
1903;27-232. doi 10.2307/2400049

Ronquist F., Teslenko M., van der Mark P., Ayres D.L., Darling A.,
Höhna S., Larget B., Liu L., Suchard M.A., Huelsenbeck J.P.
MrBayes 3.2: efficient Bayesian phylogenetic inference and model
choice across a large model space. Syst Biol. 2012;61(3):539-542.
doi 10.1093/sysbio/sys029Rozas J., Sánchez-DelBarrio J.C., Messeguer X., Rozas R. DnaSP, DNA
polymorphism analyses by the coalescent and other methods. Bioinformatics.
2003;19(18):2496-2497. doi 10.1093/bioinformatics/btg359

Ruhlman T.A., Jansen R.K. The plastid genomes of flowering plants.
Methods Mol Biol. 2014;1132:3-38. doi 10.1007/978-1-62703-
995-6_1

Souza U.J.B., Nunes R., Targueta C.P., Diniz-Filho J.A.F., Telles M.P.C.
The complete chloroplast genome of Stryphnodendron adstringens
(Leguminosae – Caesalpinioideae): comparative analysis with related
mimosoid species. Sci Rep. 2019;9(1):14206. doi 10.1038/
s41598-019-50620-3

Srivastav M., Clement W.L., Landrein S., Zhang J., Howarth D.G.,
Donoghue M.J. A phylogenomic analysis of Lonicera and its bearing
on the evolution of organ fusion. Am J Bot. 2023.110(4):e16143.
doi 10.1002/ajb2.16143

Taldybay A., Aidarbayeva D., Kurmantayeva A., Mussaev K., Amanbekova
D., Joltukova B. Medicinal plants in the flora of Zhetysu
Alatau, Zhetysu region, Kazakhstan. Casp J Environ Sci. 2024;
22(3):567-579. doi 10.22124/CJES.2024.7831

Tang D., Lin Y., Wei F., Quan C., Wei K., Wei Y., Cai Z., Kashif M.H.,
Miao J. Characteristics and comparative analysis of Mesona chinensis
Benth chloroplast genome reveals DNA barcode regions for
species identification. Funct Integr Genomics. 2022;22:467-479. doi
10.1007/s10142-022-00846-8

Varlashchenko L., Balabak A., Mamchur V., Polishchuk V. Application
of introduced representatives of Lonicera pileata Oliv. in landscaping
of the Right-Bank Forest-Steppe of Ukraine. Grassroots J Nat
Res. 2021;4(3):34-41. doi 10.33002/nr2581.6853.040304

Vdovina T.A. Biochemical evaluation of fruits of promising forms of
Altai honeysuckle (Lonicera altaica Pall.), introduced in the conditions
of the Astana Botanical Garden. Probl Bot South Sib Mongol.
2019;18(1):556-560. doi 10.14258/pbssm.2019117 (in Russin)

Vdovina T.A., Lagus O.A., Isakova E.A., Vinokurov A.A. State of coenopopulations
of wild berry plants in the territory of Kazakhstan
Altai. Bull Karaganda Univ Biol Med Geogr Ser. 2024;11629(4):
129-134. doi 10.31489/2024BMG4/129-134

Wang G.Q., Morales-Briones D.F., Landis J.B., Wang H.X., Wang H.F.
Progress in molecular systematics of Caprifoliaceae. Taxon. 2024;
74(1):5-12. doi 10.1002/tax.13279

Wang H.X., Liu H., Moore M.J., Landrein S., Liu B., Zhu Z.X.,
Wang H.F. Plastid phylogenomic insights into the evolution of the
Caprifoliaceae s.l. (Dipsacales). Mol Phylogenet Evol. 2020;142:
106641. doi 10.1016/j.ympev.2019.106641

Wang L., Li F., Zhao K., Yang J., Sun H., Cui X., Dong W., Li E., Wang N.
Comparative plastomes sheds light on phylogeny of Weigela. Front
Plant Sci. 2024;15:1487725. doi 10.3389/fpls.2024.1487725

Wang X.R., Szmidt A.E. Molecular markers in population genetics of
forest trees. Scand J For Res. 2001;16(3):199-220. doi 10.1080/
02827580118146

Wen J. Evolution of Eastern Asian–Eastern North American biogeographic
disjunctions: a few additional issues. Int J Plant Sci. 2001;
162(S6):S117-S122. doi org/10.1086/322940

Wu L., Cui Y., Wang Q., Xu Z., Wang Y., Lin Y., Song J., Yao H.
Identification and phylogenetic analysis of five Crataegus species
(Rosaceae) based on complete chloroplast genomes. Planta. 2021;
254(1):14. doi 10.1007/s00425-021-03667-4

Xia C., Wang M., Guan Y., Li Y., Li J. Comparative analysis of complete
chloroplast genome of ethnodrug Aconitum episcopale and insight
into its phylogenetic relationships. Sci Rep. 2022;12:9439. doi
1038/s41598-022-13524-3

Xia Q., Zhang H., Lv D., El-Kassaby Y.A., Li W. Insights into phylogenetic
relationships in Pinus inferred from a comparative analysis
of complete chloroplast genomes. BMC Genomics. 2023;24:346. doi
10.1186/s12864-023-09439-6

Xing W., Yu W., Kong Y., Ren X., Zhu L., Li Q., Yang Y., Cheng Y.,
Wang H. Intraspecific chloroplast genome genetic polymorphism of
Pinellia ternata (Xi Junecry) and its revelation of a single origin in
phylogeny. Genes. 2024;15(12):1638. doi 10.3390/genes15121638

Yang C., Zhang N., Wu S., Jiang C., Xie L., Yang F., Yu Z. A comparative
analysis of the chloroplast genomes of three Lonicera medicinal
plants. Genes. 2023;14(3):548. doi 10.3390/genes14030548

Yang Q.R., Zhao Y.Y., Hao J.B., Li W.D. Research progress on chemical
constituents and their differences between Lonicerae japonicae
flos and Lonicerae flos. Zhongguo Zhong Yao Za Zhi. 2016;41(7):
1204-1211 (in Chinese) doi 10.4268/cjcmm20160708

Yang X.L., Sun Q.H., Morales-Briones D.F., Landis J.B., Chen D.J.,
Wang H.X., Wen J., Wang H.F. New insights into infrageneric relationships
of Lonicera (Caprifoliaceae) as revealed by nuclear ribosomal
DNA cistron data and plastid phylogenomics. J Syst Evol.
2024;62(3):333-357. doi 10.1111/jse.13014

Yermagambetova M., Abugalieva S., Turuspekov Y., Almerekova S.
Illumina
sequencing data of the complete chloroplast genome of
rare species Juniperus seravschanica (Cupressaceae) from Kazakhstan.
Data Brief. 2023;46:108866. doi 10.1016/j.dib.2022.108866

Yermagambetova M., Almerekova S., Ivashchenko A., Turuspekov Y.,
Abugalieva S. Genetic diversity of Tulipa alberti and T. greigii
populations from Kazakhstan based on application of expressed sequence
tag simple sequence repeat markers. Plants. 2024;13(18):
2667. doi 10.3390/plants13182667

Yoo H.J., Kang H.J., Song Y.S., Park E.H., Lim C.J. Anti-angiogenic,
antinociceptive and anti-inflammatory activities of Lonicera japonica
extract. J Pharm Pharmacol. 2008;60(6):779-786. doi 10.1211/
jpp.60.6.001

Zhang T., Li M., Zhu X., Li S., Guo M., Guo C., Shu Y. Comparative
chloroplast genome analysis provided adaptive evolution insights
in Medicago ruthenica. Int J Mol Sci. 2024;25:8689. doi 10.3390/
ijms25168689

Zhang X.F., Landis J.B., Wang H.X., Zhu Z.X., Wang H.F. Comparative
analysis of chloroplast genome structure and molecular dating
in Myrtales. BMC Plant Biol. 2021;21:219. doi 10.1186/s12870-
021-02985-9

Zhang Z., Zhang Y., Song M., Guan Y., Ma X. Species identification of
Dracaena using the complete chloroplast genome as a super-barcode.
Front Pharmacol. 2019;10:1441. doi 10.3389/fphar.2019.01441

Zhao S.Y., Muchuku J.K., Liang H.Y., Wang Q.F. A complete chloroplast
genome of a traditional Chinese medicine herb, Rubia podantha,
and phylogenomics of Rubiaceae. Physiol Mol Biol Plants.
2023;29:843-853. doi 10.1007/s12298-023-01302-y

Zhu M., Feng P., Ping J., Li J., Su Y., Wang T. Phylogenetic significance
of the characteristics of simple sequence repeats at the genus level
based on the complete chloroplast genome sequences of Cyatheaceae.
Ecol Evol. 2021;11(20):14327-14340. doi 10.1002/ece3.8151

Zhu S., Liu Q., Qiu S., Dai J., Gao X. DNA barcoding: an efficient technology
to authenticate plant species of traditional Chinese medicine
and recent advances. Chin Med. 2022;17:112. doi 10.1186/s13020-
022-00655-y

